# Polymorphisms in the Cholinergic Receptors Muscarinic *(CHRM2* and *CHRM3)* Genes and Alzheimer’s Disease

**Published:** 2018

**Authors:** Lim Ya Chee, Alaistair Cumming

**Affiliations:** Pengiran Anak Puteri Rashidah Sa’adatul Bolkiah Institute of Health Sciences, Universiti Brunei Darussalam, Jalan Tungku Link, BE 1410, Gadong, Brunei Darussalam

**Keywords:** Alzheimer disease, Genes, Genetic polymorphism

## Abstract

**Background:**

Disruption of the cholinergic neurotransmitter pathway which is important for cognition, memory and learning abilities has been reported in Alzheimer’s Disease (AD) patients. The receptors involved include the Cholinergic Receptors Muscarinic (*CHRM*). *CHRM2* gene has been associated with intelligence, personality traits, substance dependence and depression. *CHRM3* has been found to heterodimerize with *CHRM2*.

**Methods:**

DNA samples from 240 AD patients with SNPs rs6962027 of *CHRM2* gene and rs7511970 of *CHRM3* gene were amplified using PCR and genotyped using Restriction Fragment Length Polymorphism (RFLP). Chi-squared test was done to check if the genes are in Hardy-Weinberg equilibrium.

**Results and Conclusion:**

Although the results did not show significant associations, these data denote plausible interaction between TT in SNP rs6962027 in *CHRM2* gene and TT in SNP rs7511970 in *CHRM3* gene affecting AD risk. SNP rs7511970 of *CHRM3* gene may also exert an influence on late-onset AD.

## Introduction

Alzheimer’s Disease (AD) is an intricate neurodegenerative disorder of the Central Nervous System (CNS) 1. Muscarinic acetylcholine receptors (mAchRs) are G-protein coupled receptors located in neurons of the nervous system, cardiac and smooth muscles 2,3. *CHRM2* gene is involved in neuronal excitability, synaptic plasticity, release of acetylcholine and cognitive function 4,5. rs6962027 of *CHRM2* gene was reported to be involved in varying personality traits of agreeableness and conscientiousness, which may modulate molecular function of the gene or protein, and therefore AD development. *CHRM3* is well-distributed throughout the nervous system and heterodimerizes with *CHRM2* to form heterodimers 6. The C-terminal tail of *CH-RM3* has anti-apoptotic properties. The aim of this study was to investigate the association of the polymerphic variation in *CHRM2* gene (rs6962027) and *CHRM3* gene (rs7511970) in relation to early- and late-onset of Alzheimer’s disease.

## Materials and Methods

### Subjects

Samples from 240 Alzheimer’s Disease (AD) patients were collected, analyzed and divided into two categories, namely, the early-onset AD group (samples from AD patients below 65 years old) and late-onset AD group. These samples included randomly selected samples from Edinburgh, UK [named ADE samples (n=106)] and from Aberdeen [named HFR samples (n= 5)]. The other category was the late-onset AD group, randomly selected from Edinburgh [named AD samples (n=71)] and from Aberdeen [named HFR samples (n=25)]. The controls were made up of 128 healthy individuals randomly selected in Glasgow [P population (n=182)]. Aberdeen Birth Cohort (ABC) Study samples provided 196 cases.

### Genotyping

Genomic DNA samples were obtained by standard procedures from peripheral blood 7. The samples were amplified by Polymerase Chain Reaction (PCR) and genotyped using Restriction Fragment Length Polymorphism (RFLP). Two Single Nucleotide Polymorphisms (SNPs) were identified, namely, rs6962027 and rs7511970 for genotyping.

### Restriction Fragment Length Polymorphism *(RFLP)*

RFLP was done to investigate the allelic variant that each sample contained. Each sample was amplified via Polymerase Chain Reaction. Restriction enzymes of Bcc1 and BstN1 (New England Biolabs, Inc., USA) were used to detect allelic variant of the SNP rs6962027 in *CHRM2* gene and SNP rs7511970 of *CHRM3* gene, respectively.

### Data analysis

***Gene-disease association analysis:*** Comparisons of the allelic and the genotypic frequencies between different sample populations were done to determine if there was an association between controls (P populations and ABC populations) and the patient populations (ADE, AD and HFR samples).

***Chi-squared test and Hardy-Weinberg Equilibrium:*** Chi-squared test was carried out and each population is considered to be in Hardy-Weinberg Equilibrium when *χ*^2^ value was less than 3.84 (equivalent to the p-value at 0.05).

## Results

The five sample populations genotyped for *CHRM2* (SNP rs6962027) and *CHRM3* (SNP rs7511970) gene analyzed by RFLP are tabulated in table 1 in terms of genotypic and allelic frequencies of the sample populations. Table 1 shows similar genotypic and allelic frequencies for both P and ABC populations of the two *CHRM* genes. These two healthy populations are the control population. The frequencies of the major allele (A) and the minor (T) for *CHRM2* (SNP rs6962027) gene are 0.53 and 0.47, respectively. The frequencies for ADE, AD and HFR populations of *CHRM2* (SNP rs6962027) gene were almost equal (Table 1), averaging to 0.57 (for A allele) and 0.43 (for T allele) for the patient population. A reduction in the allelic frequency of the T (minor) allele is observed in the patient population. The heterozygotes (AT) and rare homozygotes (TT) occur less frequently in the patient group, showing a small decline in frequencies. A consistent trend in frequencies is observed for all the sample populations. The disparity in frequencies between A and T alleles is wider in the patient populations (ADE, AD and HFR). For SNP rs7511970 of *CHRM3* gene, the allelic frequencies for G allele and A allele are 0.53 and 0.47 correspondingly. There is a wider gap between the major allele (G) and minor allele (T) relative to other sample populations, suggesting that the G allele has an effect on late-onset AD. Nevertheless, no significant difference is found between these two populations (Tables 1 and 2).

**Table 1.** Genotyping and allele frequencies of *CHRM2* (SNP rs6962027) and *CHRM3* (SNP rs7511970) polymorphic genes

***CHRM2* gene (SNP rs6962027)**					

	**Genotype**			**Allele**	

**Sample populations**	**AA**	**AT**	**TT**	**A**	**T**

P	49 (0.27)	96 (0.53)	37 (0.20)	0.53	0.47
ABC	53 (0.27)	101 (0.52)	42 (0.21)	0.53	0.47
ADE	33 (0.33)	47 (0.47)	20 (0.20)	0.57	0.43
AD	25 (0.35)	32 (0.45)	14 (0.20)	0.58	0.42
HFR	12 (0.32)	20 (0.54)	5 (0.14)	0.59	0.41
Controls	102 (0.27)	197 (0.52)	79 (0.21)	0.53	0.47
Patients	70 (0.34)	99 (0.48)	39 (0.19)	0.57	0.43

***CHRM3* gene (SNP rs7511970)**					

	**Genotype**			**Allele**	

**Sample populations**	**GG**	**GA**	**AA**	**G**	**A**

P	50 (0.28)	92 (0.51)	37 (0.21)	0.54	0.46
ABC	60 (0.31)	89 (0.45)	47 (0.24)	0.53	0.47
ADE	26 (0.25)	59 (0.56)	21 (0.20)	0.52	0.48
AD	28 (0.39)	29 (0.41)	14 (0.20)	0.60	0.40
HFR	11 (0.30)	21 (0.57)	5 (0.13)	0.58	0.42
Controls	110 (0.29)	181 (0.48)	84 (0.22)	0.53	0.47
Patients	65 (0.30)	109 (0.51)	40 (0.19)	0.56	0.44

The control population is composed of P and ABC populations, while the patient (case) population is made up of ADE, AD and HFR populations. The whole number (such as 49 for genotype AA in P population) denotes the number of samples that possess this particular genotype in this sample in this study, whereas the number in the bracket (such as 0.27 for AA genotype in P population) denotes the genotypic frequencies. The last two columns display the allelic frequencies.

**Table 2.** Comparisons between genotypic and allelic frequencies of control (P and ABC) populations and late-onset AD patient (AD sample population with the late-onset AD samples from HFR) population for *CHRM3* gene

	**Genotype**			**Allele**	

**Sample populations**	**GG**	**GA**	**AA**	**G**	**A**
**Controls**	110 (0.29)	181 (0.48)	84 (0.22)	0.53	0.47
**Late-onset AD patients**	65 (0.30)	109 (0.51)	40 (0.19)	0.56	0.44
**Total**	175	290	124		

The G-value obtained for the genotypic frequencies and allelic frequencies from the data tabulated here are 1.15 and 0.56, respectively (NS).

The *χ*^2^ values of all sample populations of both *CH-RM2* (SNP rs6962027) and *CHRM3* (rs7511970) polymorphic genes are in Hardy-Weinberg Equilibrium. To investigate if there is any bias in terms of genotypic frequency, the genotypic frequencies are stratified in terms of males and females. Depending on the age the patient was diagnosed with AD, HFR population is categorized into either ADE for early-onset or AD for late-onset. The G-values for *CHRM2* (rs6962027) polymorphic gene were 0.17 for ADE population and 1.34 for AD population, while for the *CHRM3* (rs7511970) polymorphic gene, the G-values were 1.54 for ADE population and 0.13 for AD population (Table 3). Therefore, at 5% significance level, there is no significant difference between the male and female developing AD for both *CHRM* genes.

**Table 3.** Genotypic frequencies in males and females of AD and ADE populations

	**Male**	**Female**	**Total**
**ADE population for *CHRM2* gene**			

AA	11 (0.324)	22 (0.361)	33
AT	16 (0.471)	28 (0.459)	44
TT	7 (0.205)	11 (0.180)	18
	34	61	95
**AD population for *CHRM2* gene**			
AA	9 (0.321)	24 (0.348)	33
AT	13 (0.464)	34 (0.493)	47
TT	6 (0.214)	13 (0.188)	19
	28	71	99
**ADE population for *CHRM3* gene**			
GG	6 (0.188)	16 (0.254)	22
GA	21(0.656)	33 (0.524)	54
AA	5(0.156)	14 (0.222)	19
	32	63	95
**ADE population for *CHRM3* gene**			
GG	8 (0.286)	29 (0.408)	37
GA	14 (0.500)	30 (0.423)	44
AA	6 (0.214)	12 (0.169)	18
	28	71	99

The G-value obtained for ADE population of *CHRM2* gene is 0.17 (NS), for AD population of *CHRM2* gene is 1.34 (NS), for ADE population for *CHRM3* gene is 0.17 (NS) and for AD population for *CHRM3* gene is 1.34 (NS).

rs6962027 is located in the 3’UTR region of *CHRM2* gene. Table 4 illustrates the possible configurations of genotypes in an individual which may make up the haplotype. Table 5 shows a consistent change in TT/TT genotypic combination of the two loci (rs6962027 and rs6969811) of *CHRM2* gene. This signifies that further analysis between the TT/TT and other haplotypes genotypic combinations is needed. In addition, it is noted that the frequencies of P and ABC populations, as well as for AD and ADE population, are comparable.

**Table 4.** Possible haplotypic configurations for the genotypes at these two loci (rs6962027 and rs6969811)

**SNP rs6969811**	**SNP RS6962027**			

	**AA**	**AT**		**TT**
**TT**	AT	AT		TT
	AT	TT		TT
**TC**	AT	AT or AC		TT
	AC	TC	TT	TC
**CC**	AC	AC		TC
	AC	TC		TC

There are nine possible configurations. Note that when the individual is heterozygous for both loci, the phase of the haplotype is unknown.

**Table 5.** Summary table displaying the frequencies of the different combinations of the genotypes at the two loci on *CHRM2* gene (rs6962027 and rs6969811)

	**P population**	**ABC population**	**AD population**	**ADE population**
**AT/AT**	43 (0.24)	45 (0.26)	29 (0.29)	32 (0.32)
**AT/TT**	58 (0.33)	54 (0.32)	36 (0.36)	34 (0.34)
**TT/TT**	14 (0.08)	16 (0.09)	15 (0.15)	13 (0.13)
**AT/AC**	5 (0.03)	1 (0.01)	5 (0.05)	2 (0.02)
**AT/TC or C/TT**	31 (0.18)	33 (0.19)	11 (0.11)	13 (0.13)
**TT/TC**	19 (0.11)	17 (0.10)	4 (0.04)	5 (0.05)
**AC/AC**	2 (0.01)	0 (0)	0 (0)	0 (0)
**AC/TC**	2 (0.01)	2 (0.01)	0 (0)	0 (0)
**TC/TC**	2 (0.01)	3 (0.02)	0 (0)	2 (0.01)
	176	171	100	101

The whole numbers (such as 43 in the P population) denotes the number of individuals with that particular combination of haplotypes (in this case, AT/AT). Frequencies are calculated and stated in brackets (such as 0.24 in P population for AT/AT).

This is a classic case-control experiment with healthy and diseased individuals with Pearson *χ*^2^ test statistic to test the association by use of unrelated controls. The values obtained illustrated no association between *CHRM2* rs6262027A polymorphic variant or *CHRM3* rs-7511970T polymorphic variant and AD (Table 6).

**Table 6.** Contingency table illustrating the difference in haplotype combination frequencies between the two loci on *CHRM2* gene (rs6962027 and rs6969811) in various populations

**Populations**	**Control population**	**Patient population**	**Total**
**TT/TT**	30 (0.09)	28 (0.14)	58
**Other combinations of genotypes/haplotypes**	317 (0.91)	173 (0.86)	490
	347	201	548

The G-value obtained is 3.65 (NS) at one degree of freedom.

## Discussion

The muscarinic acetylcholine receptors are drug targets for neurodegenerative diseases. All five subtypes of the *CHRM* genes are expressed in mouse brain endothelial cells. Recent findings report that the mRNA expression of *CHRM2* and *CHRM3* corresponds to the protein concentrations 8. However, no statistically significant association was found between this polymorphic variant of the gene with AD. An interesting trend was shown between the control and patient populations. There was a decrease of AT heterozygotes and TT rare homozygotes in the patient populations, even though *CHRM2* rs6962027 polymorphic gene was observed to be in Hardy-Weinberg Equilibrium.

Statistically, rs7511970 of *CHRM3* gene is not found to be associated with AD development in this study. Yet, SNP rs7511970G polymorphic variant of this gene showed an interesting trend with respect to late-onset AD. In the late-onset AD population, an increase in GG genotype was prominent. In the AD population, the GG genotype was almost equal in frequency to GA genotype. However, statistical analysis did not yield significant result for this observation. *CHRM3* polymorphic variant gene was shown to be in Hardy-Weinberg Equilibrium. SNP rs7511970 polymorphic variant *CHRM3* gene did not show any significance with early-onset AD, but there is a possibility that this SNP rs7511970G variant may exert a weak influence on late-onset AD. As this study consists only of small sample sizes, increasing the data set and the number of SNP markers would be useful to confirm the results obtained.

The combined haplotypic analysis of the two SNPs (rs6962027 and rs6969811) of *CHRM2* gene demonstrated an increase in frequency in the cases (patients) when both SNPs were TT. At one degree of freedom, the combination of TT/TT genotype yielded a value close to the critical value at 5% significance level. Thus, the combination of TT/TT genotype may be observed to modulate AD risk and therefore the sample size of the populations in this study should be increased. Since SNP rs6969811 of *CHRM2* gene is highly significant for AD development, its interaction with another SNP may enhance or reduce the effect of AD risk. As *CHRM3* receptors were reported to heterodimerize with *CHRM2*, it is possible that *CHRM3* genes share a few roles as *CHRM2* genes and may have similar physiological effects 6.

Different polymorphic variants in these *CHRM* genes may have different levels of impact on the translational efficiency of the messenger RNA, transcribed from the respective gene, and so result in different outcomes for the disease risk. Therefore, genotyping more markers in a single gene (either *CHRM2* or *CHRM3*) would give a better picture of the effect on AD risk. In summary, this study has indicated a plausible weak effect of the combined TT in both SNPs rs6962027 and rs7511970 of *CHRM2* gene on AD risk.

## Conclusion

This study concludes that there is no association between SNP rs6962027 of *CHRM2* gene and AD, as well as between rs7511970 of *CHRM3* gene and AD. However, it is plausible that SNP rs7511970 of *CHRM*3 gene may exert an influence on late-onset AD that can only be detected with a larger sample population. As AD is a complex disease with many susceptible genes awaiting confirmation, concept of epistasis is highly applicable, as many varying genes may influence the outcome of AD confounded by the role of the environment. Therefore, tackling this puzzle of complex neurodegenerative AD is an intricate process that takes into account gene-gene interaction, gene-environment interaction and genotype-genotype.
